# Fewer breast cancers during post-treatment surveillance among patients initially staged using contrast-based imaging

**DOI:** 10.1007/s10549-026-08049-6

**Published:** 2026-08-13

**Authors:** T. W. W. Yang, A. K. Rose, J. Matheson, K. Elder, C. MacCallum, C. Nickson, G. B. Mann

**Affiliations:** 1 The Breast Service, The Royal Melbourne Hospital, Parkville, Australia; 2 Department of Surgery, The University of Melbourne, Parkville, Australia; 3 Cancer Elimination Collaboration, Sydney School of Public Health, University of Sydney, Sydney, Australia; 4 Melbourne School of Population and Global Health, The University of Melbourne, Parkville, Australia; 5 Department of Radiology, The University of Melbourne, Parkville, Australia

**Keywords:** Early breast cancer, Contrast-based imaging, Contrast-enhanced mammography, Locoregional events

## Abstract

**Purpose:**

The role of contrast-based imaging (CBI) at diagnosis in early breast cancer remains debated. More sensitive baseline staging may identify synchronous disease that would otherwise present as early ipsilateral or contralateral events. We examined whether CBI at diagnosis is associated with differences in early locoregional outcomes.

**Methods:**

A retrospective cohort study of women with early invasive breast cancer or ductal carcinoma in situ (DCIS). Patients were grouped according to whether they underwent CBI (CEM or MRI) at diagnosis. All underwent mammographic surveillance including contrast-enhanced mammography (CEM). Locoregional events from diagnosis to 30 June 2025 were identified and evaluated. Cox proportional hazards models to assess associations between CBI use and subsequent locoregional events were adjusted for age, calendar period, mammographic density (MD), and stage.

**Results:**

Among 1,106 women (594 CBI, 512 non-CBI) diagnosed between April 2015 and June 2022, there were 60 locoregional events (39 invasive, 21 DCIS). Patients who underwent CBI had fewer subsequent locoregional events. After adjustment, CBI use at diagnosis was associated with significantly lower 5-year event rates among women with invasive cancer (adjusted HR 0.50, 95% CI 0.26–0.98, *p* = 0.043), particularly those with Luminal A/B phenotypes, low MD, and with fewer contralateral events in invasive index cases (adjusted HR 0.19, 95% CI 0.05–0.71, *p* = 0.014). Features of subsequent events (including tumour grade, behaviour, and invasive size) were not found to differ between groups.

**Conclusion:**

Inclusion of CBI in local staging of early breast cancer was associated with lower rates of locoregional events over five years. These findings support broader consideration of CBI use for local staging at diagnosis.

**Supplementary Information:**

The online version contains supplementary material available at 10.1007/s10549-026-08049-6.

## Introduction

The principles of cancer treatment involve confirming the presence and nature of a cancer, defining the extent of disease and delivering treatments to minimize risk of recurrence while avoiding unnecessary side effects of therapy.

For early breast cancer, the optimal approach to local staging lacks consensus. Contrast-based imaging (CBI, including magnetic resonance imaging (MRI) and contrast-enhanced mammography (CEM)) in local staging of breast cancer remains a subject of ongoing debate and is not universally recommended for all patients [1]. It is typically reserved for individuals considered to be at higher risk of additional lesions - such as younger women, those with dense breast tissue, multifocal or lobular histology, or discordant findings on conventional imaging [1, 2].

MRI is the most extensively studied CBI modality and has been shown to detect additional foci of malignancy in 10–16% of patients [3]. Randomized trials have produced mixed results, with limited impact on re-excision rates and often an increased mastectomy rate [4–6]. Few studies have explored its association with oncological outcomes [7–10]. While a recent meta-analysis reports reductions in metachronous contralateral cancers and locoregional recurrence [7], other studies show no improvement in local relapse-free survival [8–10].

CEM is performed by acquiring dual-energy mammograms after intravenous injection of iodinated contrast. It offers higher sensitivity than 2D/3D mammography plus ultrasound in patients with dense breasts [11], and similar sensitivity and specificity to MRI [12]. In the staging setting, CEM can improve assessment of disease extent and detect otherwise occult lesions [13]. However, no studies have reported oncological outcomes comparing patients staged with and without CEM [13].

Since late 2018, the Royal Melbourne Hospital and Royal Women’s Hospital Breast Service in Melbourne, Australia has progressively implemented preoperative staging with CBI, initially in patients with invasive breast cancer and those with higher mammographic density (MD). In more recent years it has become near-universal for preoperative staging of early breast cancer or ductal carcinoma in situ (DCIS). CEM was progressively adopted as the sole surveillance modality for most women with a personal history of breast cancer (PHBC) since 2018. Our group has previously reported a series of 202 patients with screen-detected early breast cancer in which preoperative CEM identified occult malignancy in 14% and altered surgical management in 21% [14]. In a separate analysis of nearly 2,600 surveillance episodes for women with PHBC, CEM demonstrated high sensitivity, with almost half of surveillance-detected cancers identified only on contrast-enhanced views [15].

We hypothesize that more sensitive initial staging may identify and allow treatment of synchronous disease at diagnosis that would otherwise present as early ipsilateral recurrence or contralateral cancer [16]. This study evaluates whether use of CBI at diagnosis is associated with differences in early locoregional outcomes, using adjusted Cox models to compare subsequent locoregional events, ipsilateral and contralateral event rates, and details of locoregional tumour characteristics.

## Methodology

### Study design and population

This was a retrospective cohort study conducted at the Breast Service of the Royal Melbourne Hospital and Royal Women’s Hospital, Melbourne, Australia. We identified women diagnosed with early breast cancer between April 2015 and June 2022 who underwent initial surveillance with CEM between June 2016 and December 2022. Women in both groups underwent annual surveillance imaging. CEM was selectively used for surveillance from June 2016 and, from late 2018, was progressively adopted as the default surveillance modality. Consequently, women who underwent CBI at diagnosis generally received CEM from their first surveillance round, whereas women in the non-CBI group, particularly those diagnosed earlier in the study period, often underwent one or more rounds of conventional mammographic surveillance before transitioning to CEM. All surveillance episodes were evaluated from diagnosis through to 30 June 2025.

### Study groups

Patients were categorized according to whether they underwent CBI - either CEM or MRI – for local staging prior to definitive breast surgery. Those without CBI at diagnosis served as the comparison group. To ensure a consistent interval to capture early post-treatment surveillance, only women who underwent their first surveillance CEM within three years of their index breast cancer diagnosis were included.

### Data collection and outcome measures

Clinical, radiologic, and histopathologic data were obtained from the hospital records, and were cross-checked for completeness. Collected variables included patient demographics, tumour characteristics at index diagnosis, MD assessed from the first surveillance CEM episode (automated volumetric analysis using VOLPARA Health Technologies) and details of subsequent locoregional events.

Subsequent locoregional events were characterised by laterality, pathological subtype, mode of presentation, and tumour size, grade, and stage.

The primary analysis assessed the incidence of locoregional events according to CBI use at diagnosis, using Cox proportional hazards models. Models were adjusted for age at diagnosis, calendar period, stage of index diagnosis, and MD. Secondary analyses examined tumour characteristics (behaviour, grade and size) of locoregional events and separately assessed contralateral events. Patients were censored at the earliest of a subsequent locoregional event, death, or end of the study period.

### Data reporting and statistical analysis

Baseline characteristics of the study cohort and outcomes of locoregional events during follow-up were summarised according to use of CBI at diagnosis. Continuous variables were reported as means or medians (with interquartile ranges), and categorical variables were reported using frequencies and percentages, testing for differences according to CBI at diagnosis using appropriate parametric and non-parametric tests (namely, Fisher’s exact or rank sum for categorical variables, and t-tests for continuous variables).

**Fig. 1:**
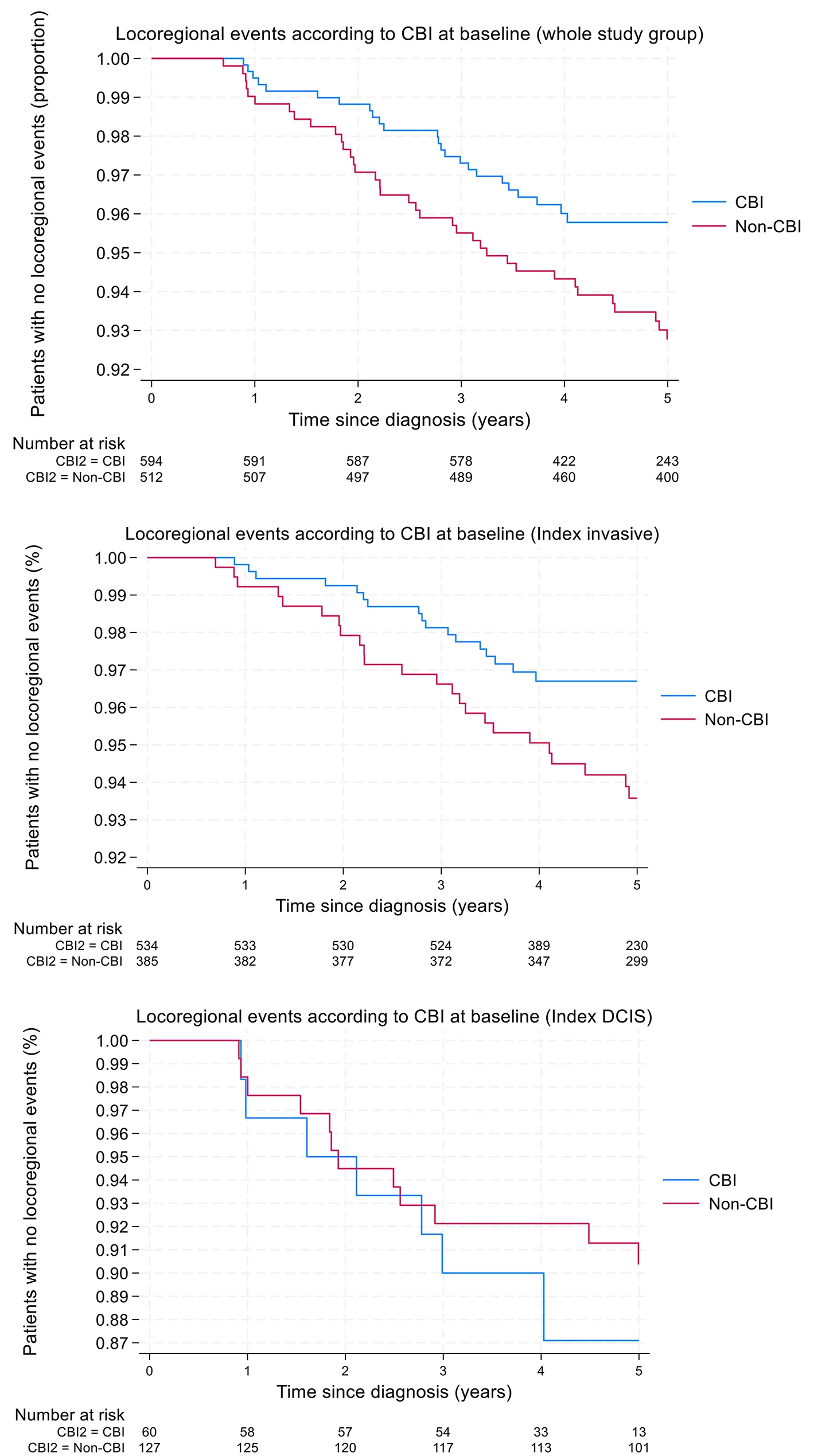
Observed locoregional event-free survival according to CBI use for local staging at diagnosis

The association between CBI at diagnosis and locoregional event rates within 5 years was evaluated using Cox proportional hazards models to account for differences in follow-up between women. Models were fitted for the whole study group, separately for invasive and DCIS index cases, and for patients with Luminal A/B (ER/PR+, HER2-/+) index cancers. For invasive index cancers, contralateral events were analysed separately using similarly adjusted Cox models. Time at risk commenced at index cancer diagnosis, with censoring at death, distant metastasis, last negative surveillance, or 5 years (whichever came first). The hazards model was adjusted for age, calendar period, index cancer stage and MD from the first surveillance CEM episode. Unadjusted and adjusted hazard ratios were reported, and predicted locoregional event-free survival curves plotted. Univariate hazards models were also reported for all covariates in the adjusted models. (Supplementary Tables 1 and Fig. 1)

## Results

### Study population and baseline characteristics

Between April 2015 and June 2022, 1,106 women met the inclusion criteria, including 919 with invasive breast cancer and 187 with DCIS. The median age at diagnosis was 66 (range 34–98). Baseline characteristics according to use of CBI at diagnosis are summarized in Table 1.

**Table 1:** Baseline patient characteristics according to CBI use at diagnosis

		CBI at diagnosis			*p*-value*
		CBI	Non-CBI	Total	
Patients	N	594 (53.7%)	512 (46.3%)	1,106 (100.0%)	
Age at diagnosis	< 50	59 (9.9%)	21 (4.1%)	80 (7.2%)	< 0.001
	50–69	326 (54.9%)	304 (59.4%)	630 (57.0%)	
	70+	209 (35.2%)	187 (36.5%)	396 (35.8%)	
	Age (median (range))	66 (37–98)	65 (34–94)	66 (34–98)	< 0.001
CBI modality at diagnosis	CEM	355 (59.8%)	N/A	355 (59.8%)	N/A
	MRI	202 (34.0%)	N/A	202 (34.0%)	
	Both	37 (6.2%)	N/A	37 (6.2%)	
Diagnosis date (calendar year)	2014–2018	145 (24.4%)	316 (61.7%)	461 (41.7%)	< 0.001
	2019–2022	449 (75.6%)	196 (38.3%)	645 (58.3%)	
MD	A/B	281 (47.3%)	279 (54.5%)	560 (50.6%)	0.017
	C/D	313 (52.7%)	233 (45.5%)	546 (49.4%)	
Index tumour behaviour	Invasive	534 (89.9%)	385 (75.2%)	919 (83.1%)	< 0.001
	DCIS	60 (10.1%)	127 (24.8%)	187 (16.9%)	
Index invasive size	Median (range)	14 (0-293)	13 (0-150)	13 (0-293)	0.370
	≤ 2 cm	376 (70.4%)	260 (67.5%)	636 (69.2%)	0.638
	2–5 cm	138 (25.8%)	110 (28.6%)	248 (27.0%)	
	> 5 cm	20 (3.7%)	15 (3.9%)	35 (3.8%)	
Index invasive grade	1	114 (21.3%)	67 (17.4%)	181 (19.7%)	< 0.001
	2	273 (51.1%)	163 (42.3%)	436 (47.4%)	
	3	133 (24.9%)	136 (35.3%)	269 (29.3%)	
	Not available	14 (2.6%)	19 (4.9%)	33 (3.6%)	
Index invasive phenotype	ER/PR+ HER2-	446 (83.5%)	289 (75.1%)	735 (80.0%)	0.018
	ER/PR/HER2+	39 (7.3%)	43 (11.2%)	82 (8.9%)	
	ER/PR- HER2+	13 (2.4%)	15 (3.9%)	28 (3.0%)	
	TNBC	36 (6.7%)	38 (9.9%)	74 (8.1%)	
Index invasive nodal involvement	Positive	128 (24.0%)	95 (24.7%)	223 (24.3%)	0.797^
	Negative	383 (71.7%)	273 (70.9%)	656 (71.4%)	
	Not available	23 (4.3%)	17 (4.4%)	40 (4.4%)	
Index invasive stage	1	324 (60.7%)	217 (56.3%)	541 (58.9%)	0.369
	2	179 (33.5%)	140 (36.4%)	319 (34.7%)	
	3	31 (5.8%)	28 (7.3%)	59 (6.4%)	
Index DCIS grade	Low Grade	5 (8.3%)	14 (11.0%)	19 (10.2%)	0.599^
	Intermediate Grade	20 (33.3%)	48 (37.8%)	68 (36.4%)	
	High Grade	29 (48.3%)	52 (40.9%)	81 (43.3%)	
	Not available	6 (10.0%)	13 (10.2%)	19 (10.2%)	
Index surgery type**	Mastectomy	116 (19.5%)	87 (17.0%)	203 (18.4%)	0.311
	BCS	478 (80.5%)	425 (83.0%)	903 (81.6%)	
Radiation therapy***	Yes	375 (72.1%)	279 (75.6%)	654 (73.6%)	0.244
	No	145 (27.9%)	90 (24.4%)	235 (26.4%)	
Endocrine therapy	Yes	454 (89.0%)	310 (84.9%)	764 (87.3%)	0.073
	No	56 (11.0%)	55 (15.1%)	111 (12.7%)	
Chemotherapy	Yes	174 (33.7%)	152 (40.9%)	326 (36.7%)	0.028
	No	343 (66.3%)	220 (59.1%)	563 (63.3%)	

N/A = Not applicable. MD = Mammographic density. BCS = breast conserving surgery *Test for difference according to CBI at diagnosis. ^Test excludes missing values **In two patients in the CBI group who underwent bilateral surgery (BCS on one side and mastectomy on the other), index surgery type was classified according to the more extensive procedure (mastectomy) ***Refer to supplementary Table 3 for expanded treatment details

**Fig. 2:**
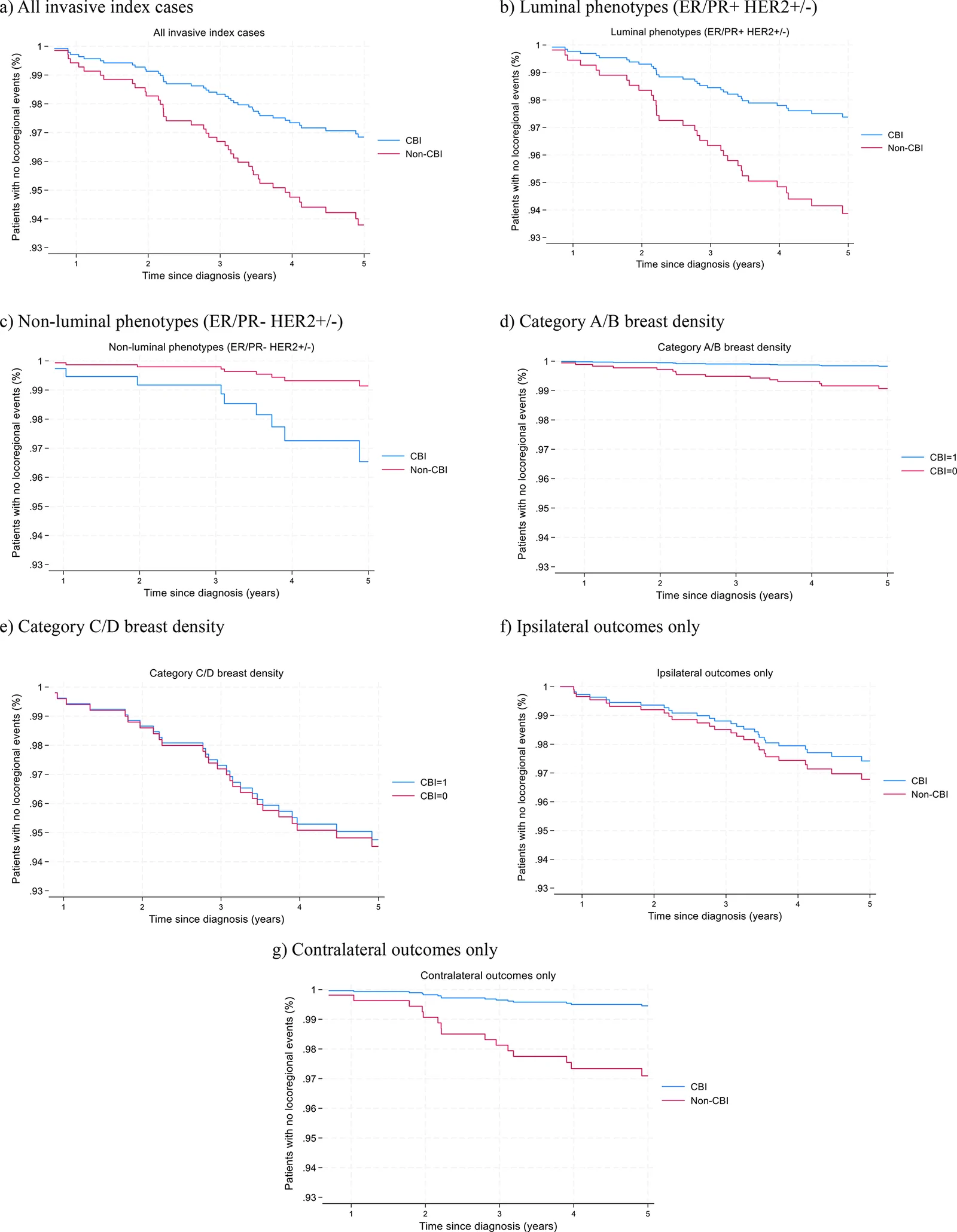
Adjusted-predicted hazard curves for locoregional events (index invasive only), according to CBI use at staging. All figures adjusted for age group, calendar period, and stage. **a**-**c** and f-g also adjusted for breast density

Among invasive cancers, median tumour size was 13 mm, 24.3% were node positive, and pathological stage was Stage 1, 2 and 3 in 58.9%, 34.7% and 6.4% respectively. Most tumours had Luminal phenotype (ER/PR+, HER2-/+) (88.9%), while 3.0% were HER2 enriched (ER/PR-, HER2+) and 8.1% were triple-negative. Surgical management consisted of breast-conserving surgery (BCS) in 81.4% and mastectomy in 18.6%. Adjuvant radiotherapy (RT) was administered to 79.0% of women treated with BCS and 36.8% of those undergoing mastectomy. Chemotherapy was used in 35.5% of women overall, while endocrine therapy was prescribed in 83.1% overall, and 92.0% of women with ER-positive invasive disease.

Among women with DCIS, the median lesion size was 10 mm and 43.3% had high grade disease. Most were managed with BCS (84.0%), of whom 49.7% received adjuvant RT, while 16.0% underwent mastectomy. Endocrine therapy was recommended in 62.0% of women with DCIS.

CBI at diagnosis was performed in 594 women (53.7%), comprising CEM in 355, MRI in 202 and both modalities in 37. Compared with the non-CBI group, CBI use was more frequent among women diagnosed after 2019 (75.6% vs. 38.3%, *p* < 0.001), in those with invasive cancer (89.9% vs. 75.2%, *p* < 0.001) and in those with higher MD (MD C/D: 52.7% vs. 45.5%, *p* = 0.017). By pathological stage, CBI was used in 32.1% of women with Stage 0 disease, 59.9% with Stage 1, 56.1% with Stage 2, and 52.5% with Stage 3 disease.

### Follow-up and locoregional events

There was a total of 5,031 person-years of follow-up up until June 2025 with a mean follow-up of 4.5 years per patient as shown in Table 2. Surveillance prior to 2019 was primarily performed using conventional imaging (mammography with or without ultrasound), transitioning to CEM from 2019 onwards [17]. Most women in both groups underwent at least three surveillance CEM examinations (CBI: 545/594, 91.8%; non-CBI: 440/512, 85.9%).

**Table 2:** Locoregional events during follow-up according to CBI use for local staging at diagnosis

Outcome	Measure	CBI	No-CBI	All patients	*p*-value*
Patients	N	594	512	1,106	
**Follow-up**					
Surveillance episodes	Total	2,116	1,754	3,870	< 0.002
	Mean (SD) per patient	3.6 (1.0)	3.4 (1.1)	3.5 (1.1)	0.034
	Median (range)	4 (1–6)	4 (1–6)	4 (1–6)	0.034
Time to first CEM surveillance (years)	Median (range)	0.9 (0.2-3.0)	1.2 (0.1–3.0)	1.0 (0.1-3.0)	< 0.001
Duration of follow-up	Total (person-years)	2,616	2,415	5,031	
	Average per patient (years)	4.4	4.7	4.5	
**Locoregional events**					
Total	N (%)	24	36	60	-
Morphology	Invasive	18 (75.0%)	21 (58.3%)	39 (65.0%)	0.185
	DCIS	6 (25.0%)	15 (41.7%)	21 (35.0%)	
Laterality	Ipsilateral***	18 (75.0%)	22 (61.1%)	40 (66.7%)	0.264
	Contralateral	6 (25.0%)	14 (38.9%)	20 (33.3%)	
Mode of presentation	Surveillance-detected	21 (87.5%)	35 (97.2%)	56 (93.3%)	0.139
	Interval cancer	3 (12.5%)	1 (2.8%)	4 (6.7%)	

MD = mammographic density *Test for difference according to CBI at diagnosis. ^Test excludes missing values. **MD measured from first surveillance CEM ***Includes cases with index bilateral disease

A total of 60 locoregional events were observed, including 39 invasive and 21 DCIS events, with 24 occurring in the CBI group and 36 in the non-CBI group. Fifty-six events were detected during routine surveillance and four were interval cancers. There were 40 ipsilateral events (18 CBI group, 22 non-CBI group) and 20 contralateral events (6 CBI group, 14 non-CBI group). Locoregional events occurred more frequently following DCIS than invasive index diagnoses (8.6% vs. 4.8%).

Tumour characteristics of locoregional events are detailed in Table 3, while their timing and contribution of contrast to detection are summarized in Supplementary Table 2. There were no significant differences between CBI and non-CBI groups in event morphology, size, grade, nodal involvement, phenotype or stage.

**Table 3:** Tumour characteristics of locoregional events

		CBI at diagnosis			*p*-value
		CBI	Non-CBI	Total	
**Invasive**					
Size (mm)	Mean (SD)	23.1 (25.8)	14.2 (10.7)	18.4 (19.4)	0.154
	≤ 2 cm	11 (61.1%)	16 (76.2%)	27 (69.2%)	0.258
	2–5 cm	5 (27.8%)	5 (23.8%)	10 (25.6%)	
	> 5 cm	2 (11.1%)	0 (0.0%)	2 (5.10%)	
Grade	1	0 (0.0%)	4 (19.0%)	4 (10.3%)	0.107
	2	10 (55.6%)	8 (38.1%)	18 (46.2%)	
	3	8 (44.4%)	7 (33.3%)	15 (38.5%)	
	unknown	0 (0.0%)	2 (9.5%)	2 (5.1%)	
Phenotype	ER/PR+ HER2-	10	13	23	
	ER/PR/HER2+	3	1	4	
	ER/PR- HER2+	1	3	4	
	TNBC	4	3	7	
	Not available	-	1	1	
Nodal involvement	Positive	4 (22.2%)	3 (14.3%)	7 (18.0%)	0.419^
	Negative	10 (55.6%)	15 (71.4%)	25 (64.1%)	
	Unknown*	4 (22.2%)	3 (14.3%)	7 (18.0%)	
TNM Stage	1	10 (55.6%)	15 (66.7%)	24 (61.5%)	0.463^
	2a	6 (33.3%)	6 (28.6%)	12 (30.8%)	
	2b	0	0	0	
	3	1 (5.6)	0 (0.0%)	1 (2.6%)	
	4	1 (5.6%)	0 (0.0%)	1 (2.6%)	
**DCIS**					
Size (mm)	≤ 2 cm	3 (50.0%)	10 (71.4%)	13 (65.0%)	0.393
	2–5 cm	3 (50.0%)	3 (21.4%)	6 (30.0%)	
	> 5 cm	0 (0.0%)	1 (7.1%)	1 (5.0%)	
Grade	Low	0 (0%)	0 (0%)	0 (0%)	0.692^
	Intermediate	2 (33.3%)	6 (40.0%)	8 (38.1%)	
	High	4 (66.7%)	7 (53.3%)	12 (57.1%)	
	Not available	0 (0.0%)	1 (6.7%)	1 (6.7%)	

*Nodal status assumed to be negative where it was unknown

### Locoregional event rates and survival analyses

Observed locoregional event-free survival according to CBI use at diagnosis is shown in Fig. 1. For the whole cohort, the unadjusted association between CBI and locoregional recurrences showed a trend towards lower event rates but did not reach statistical significance (unadjusted HR 0.61, 95% CI 0.36–1.02, *p* = 0.061).

Event rates and hazard ratios accounting for duration of follow-up are summarized in Table 4. Among all patients (index invasive and DCIS), locoregional events rates were lower in the CBI group (9.2 vs. 14.9 per 1,000 person-years), although this was not statistically significant after adjustment for age, calendar period, MD and stage (adjusted HR 0.66, 95% CI 0.37–1.17, *p* = 0.153). In contrast, among patients with invasive index cancers, CBI at diagnosis was associated with a significantly reduced risk of locoregional events after adjustment (adjusted HR 0.50, 95% CI 0.26–0.98, *p* = 0.043; Fig. 2a).

**Table 4:** Rates and hazards of locoregional events according to CBI use for local staging of index diagnosis (CBI vs. non-CBI), accounting for duration of follow-up

	Analysis	Number of locoregional events			Locoregional event rate per 1,000 person years (95% CI)			Unadjusted HR (95% CI), p-value	Adjusted HR (95% CI), p-value
		CBI	Non-CBI	Total	CBI	Non-CBI	Total		
Index invasive + DCIS	All patients	24	36	60	9.2 (6.2–13.7)	14.9 (10.8–20.7)	11.9 (9.3–15.4)	0.61 (0.36–1.02), *p* = 0.061	0.66 (0.37–1.17), *p* = 0.153
	MD Category A/B*	7	16	23	5.6 (2.7–11.8)	12.1 (7.4–19.8)	9.0 (6.0–13.5)	0.45 (0.19–1.10), *p* = 0.080	0.41 (0.16–1.05), *p* = 0.063
	MD Category C/D*	17	20	37	12.4 (7.7–19.9)	18.3 (11.8–28.3)	15.0 (10.9–20.7)	0.68 (0.35–1.30), *p* = 0.242	0.93 (0.45–1.94), *p* = 0.844
	Contralateral LR events	6	14	20	2.3 (1.0–5.1)	5.8 (3.4–9.8)	4.0 (2.6–6.2)	0.39 (0.15–1.01), *p* = 0.053	0.37 (0.13–1.05), *p* = 0.062
	Ipsilateral LR events	18	22	40	6.9 (4.3–10.9)	9.1 (6.0–13.8)	8.0 (5.8–10.8)	0.75 (0.40–1.40), *p* = 0.369	0.84 (0.42–1.69), *p* = 0.622
Index invasive cancers only	**All**	**17**	**24**	**41**	**7.2 (4.4–11.5)**	**13.2 (8.8–19.7)**	**9.8 (7.2–13.3)**	**0.54 (0.29–1.01), p = 0.052**	**0.50 (0.26–0.98), p = 0.043**
	**MD Category A/B**	**3**	**12**	**15**	**2.7 (0.9–8.3)**	**12.4 (7.0–21.8)**	**7.2 (4.3–11.9)**	**0.22 (0.06–0.76), p = 0.018**	**0.18 (0.05–0.67), p = 0.010**
	MD Category C/D	14	12	26	11.2 (6.6–18.9)	14.1 (8.0–24.8)	12.3 (8.4–18.1)	0.79 (0.36–1.70), *p* = 0.541	0.96 (0.41–2.25), *p* = 0.918
	Stage 1/2a	15	20	35	7.6 (4.6–12.6)	13.0 (8.4–20.2)	10.0 (7.2–13.9)	0.57 (0.29–1.12), *p* = 0.104	0.50 (0.24–1.02), *p* = 0.055
	Stage 2b/3	2	4	6	5.0 (1.3–20.0)	14.0 (5.3–37.3)	8.7 (3.9–19.5)	0.38 (0.07–2.12), *p* = 0.272	0.73 (0.10–5.05), *p* = 0.747
	**Lower-risk phenotype (ER/PR+, HER2-/+)**	**13**	**19**	**32**	**6.0 (3.5–10.3)**	**12.1 (7.7–19.0)**	**8.6 (6.1–12.1)**	**0.49 (0.24–0.99), p = 0.046**	**0.42 (0.20–0.89), p = 0.024**
	Higher-risk phenotypes (ER/PR- HER2+/-)	4	5	9	19.1 (7.2–51.0)	19.9 (8.3–47.7)	19.5 (10.2–37.5)	1.05 (0.27–3.78), *p* = 0.993	1.57 (0.31–7.88), *p* = 0.581
	**Contralateral LR events**	**3**	**12**	**15**	**1.3 (0.4–3.9)**	**6.6 (3.7–11.6)**	**3.6 (2.2–5.9)**	**0.19 (0.05–0.67), p = 0.010**	**0.19 (0.05–0.71), p = 0.014**
	Ipsilateral LR events	14	12	26	5.9 (3.5–9.9)	6.6 (3.7–11.6)	6.2 (4.2–9.1)	0.89 (0.41–1.92), *p* = 0.769	0.80 (0.35–1.83), *p* = 0.597
Index DCIS only^	All	7	12	19	29.1 (13.9–61.2)	20.2 (11.5–35.6)	22.8 (14.5–35.8)	1.40 (0.55–3.59), *p* = 0.484	1.41 (0.48–4.17), *p* = 0.532
	MD Category A/B	4	4	8	33.3 (12.5–88.6)	11.4 (4.3–30.4)	17.0 (8.5–34.0)	2.45 (0.61–9.78), *p* = 0.206	1.75 (0.33–9.19), *p* = 0.511
	MD Category C/D	3	8	11	25.1 (8.1–77.8)	33.0 (16.5–65.9)	30.4 (16.8–54.8)	0.78 (0.21–2.97), *p* = 0.716	0.99 (0.23–4.31), *p* = 0.990
	Contralateral LR events	3	2	5	12.5 (4.0–38.8)	3.4 (0.8–13.5)	6.0 (2.5–14.4)	3.57 (0.59–21.6), *p* = 0.166	3.73 (0.47–29.71), *p* = 0.214
	Ipsilateral LR events	4	10	14	16.7 (6.3–44.5)	16.9 (9.1–31.3)	16.8 (10.0–28.4)	0.96 (0.30–3.09), *p* = 0.944	0.96 (0.26–3.59), *p* = 0.948

LR = locoregional. MD = mammographic density. Models adjusted for age group, calendar period, MD and stage, except for ^ adjusted for age group, calendar period and MD. *MD measured from first surveillance CEM. Analysis includes all LR events unless stated otherwise

When stratified by MD, the association between CBI use at diagnosis and locoregional event risk differed according to MD category (Table 4). The locoregional event rate was numerically higher in those with high MD (MD C/D) compared to those with low MD (MD A/B) (37 vs. 23 per 1,000 person-years). Among patients with low MD, locoregional event rates were lower in the CBI group compared with the non-CBI group (5.6 vs. 12.1 per 1,000 person-years), with a trend towards reduced risk after adjustment (adjusted HR 0.41, 95% CI 0.16–1.05, *p* = 0.063). Somewhat surprisingly, no significant CBI association was observed among patients with high MD (MD C/D) (adjusted HR 0.93, 95% CI 0.45–1.94, *p* = 0.844). This differential effect was more pronounced among patients with invasive index cancers, in whom CBI use was associated with a significantly reduced risk of locoregional events in those with low MD (adjusted HR 0.18, 95% CI 0.05–0.67, *p* = 0.010; Fig. 2d), whereas no significant association was observed in patients with high MD (adjusted HR 0.96, 95% CI 0.41–2.25, *p* = 0.918; Fig. 2e). Among patients with index DCIS, no significant association between CBI use and locoregional events was observed in either MD subgroup (low MD adjusted HR 1.75, 95% CI 0.33–9.19, *p* = 0.511, high MD adjusted HR 0.99, 95% CI 0.23–4.31, *p* = 0.990).

When stratified by stage, a stronger inverse association was observed in patients with earlier-stage disease. For Stage 1/2a cancers, locoregional event rates were lower in the CBI group (7.6 vs. 13.0 per 1,000 person-years), with a trend towards reduced risk after adjustment (adjusted HR 0.50, 95% CI 0.24–1.02, *p* = 0.055). In contrast, no significant association was observed for patients with Stage 2b/3 disease (adjusted HR 0.73, 95% CI 0.10–5.05, *p* = 0.747).

Stratification by tumour phenotype demonstrated that the association between CBI use and reduced locoregional event risk was more pronounced in patients with luminal phenotypes (ER/PR+, HER2+/-), in whom CBI at diagnosis was associated with approximately halved event rates (adjusted HR 0.42, 95% CI 0.20–0.89, *p* = 0.024; Fig. 2b). No significant association was observed in other phenotypes (ER/PR-, HER2+/-) (adjusted HR 1.57, 95% CI 0.31–7.88, *p* = 0.581; Fig. 2c).

CBI use was also associated with a significantly lower rate of contralateral locoregional events among patients with invasive index cancers (adjusted HR 0.19, 95% CI 0.05–0.71, *p* = 0.014; Table 4; Fig. 2g). No significant association between CBI use and locoregional events was observed for patients with index DCIS (adjusted HR 1.41, 95% CI 0.48–4.17, *p* = 0.532).

## Discussion

This study demonstrates an association between the use of CBI at diagnosis and a reduced incidence of locoregional events, with statistically significant effects observed in specific, clinically relevant subgroups. These findings suggest that more sensitive baseline imaging may influence downstream oncological outcomes, potentially by identifying otherwise occult disease at the time of diagnosis.

Minimizing locoregional events remains a central objective in the management of early breast cancer. Historically, locoregional recurrence rates (LRR) approached 40% following BCS alone and approximately 15% with the addition of adjuvant RT [18]. With advances in various aspects of treatment, these outcomes have substantially improved, and contemporary randomized trials in Stage 1 ER-positive disease report LRR rates at 10 years of approximately 10% without RT and 1–2% with RT [19, 20].

Considerable effort has gone in to identifying factors associated with LRR, including tumour size, nodal involvement [21, 22], histologic grade [21–23], adverse molecular subtypes (triple-negative and HER2-positive) [24], and more recently higher MD [25]. The association between higher MD and LRR has been postulated to reflect masking of occult disease on conventional imaging, with subsequent manifestation as locoregional events [16, 22]. Despite this, relatively little attention has been paid to whether the choice of baseline imaging modality influences LRR risks.

CBI is more sensitive than conventional mammography and ultrasound and has been shown to identify additional foci of disease in up to 10–15% of patients at diagnosis [3]. Randomized trials evaluating preoperative MRI have primarily focussed on surgical outcomes, with mixed results including higher mastectomy rates in some studies and lower re-excision rates in others [4–6]. Fewer studies have evaluated oncological outcomes [7–10], and while meta-analyses such as that by Eisen et al. suggest a favourable association between MRI at diagnosis and recurrence-related endpoints [7], this remains an area of ongoing debate.

We leveraged the progressive implementation of near-routine CBI for local staging within our breast service to examine its association with early locoregional outcomes. In this cohort, CBI use at diagnosis was associated with lower rates of locoregional events overall, although this did not reach statistical significance for the entire study population after adjustment. However, among patients with index invasive cancers, CBI was independently associated with a significantly reduced risk of locoregional events within 5 years. This corresponded to an approximate 50% relative reduction in locoregional events, a magnitude of effect comparable, in proportional terms, to that reported with adjuvant radiotherapy following breast-conserving surgery [26]. The association was particularly evident in patients with earlier stage disease and in those with luminal phenotypes, whereas no significant association was observed in patients with ER negative phenotypes or in those with index DCIS. The lack of a significant association among patients with index DCIS is likely related to the higher incidence of non-enhancing DCIS compared with invasive disease [27], but may also reflect the smaller size of the DCIS subgroup and limited event numbers. Adjusted hazards models were used to account for key clinicopathologic factors, reflecting the intent to identify patient groups in whom CBI at diagnosis may be most informative in clinical settings where contrast-based staging is not routinely employed.

The finding that CBI at diagnosis confers a greater relative benefit in patients with earlier-stage luminal disease suggests that these cancers may be truly localised at presentation. In this setting, identification and treatment of otherwise occult foci is likely to be particularly impactful. In contrast, in higher-stage disease – where both local extent and the likelihood of systemic dissemination are greater – the incremental benefit of more sensitive local staging appears attenuated [16, 28, 29].

While higher MD was associated with greater use of CBI, a clinically relevant reduction in locoregional events with CBI was evident only among patients with invasive cancer with lower MD. Notably, no significant association was observed in the higher MD subgroup, despite their overall higher locoregional event rates. This pattern is somewhat counter-intuitive, as one might expect the greatest incremental benefit of contrast-based techniques in denser breasts, where masking on conventional imaging is more pronounced [16, 22]. These findings need to be validated in separate series before being applied to practice, but they do strongly suggest a benefit to preoperative CBI in those with lower MD.

The differential effect observed by tumour phenotype is also biologically plausible, although the small numbers warrant cautious interpretation. Systemic therapy does not generally result in a pathological complete response in luminal cancers [30]. Therefore, the identification and local treatment of occult disease may translate into meaningful reductions in early locoregional events. In contrast, for biologically aggressive phenotypes such as triple-negative or HER2-enriched disease, systemic therapy may dominate outcomes [30, 31], and additional local disease detection may have less clinical impact. Longer follow-up to determine the rate of locoregional events once adjuvant ET is complete will be important, particularly as most patients with luminal disease remain on ET throughout the first five years after diagnosis [32, 33]. The persistence of LRR beyond five years in trials such as PRIME II [19] and CALGB 9343 [20] underscores the need for extended follow-up.

Notably, the most pronounced difference associated with CBI use was observed for contralateral events among patients with index invasive cancers. This supports the hypothesis that a substantial proportion of early contralateral recurrences represent occult synchronous disease. The absence of a significant ipsilateral association may be explained by the effects of adjuvant therapy. Ipsilateral occult disease is typically subject to adjuvant RT and systemic therapy which together may eradicate residual microscopic disease [26, 34]. In contrast, occult contralateral disease is treated with systemic therapy alone [35] and is therefore less likely to be eradicated, and present as an early contralateral event if not identified at baseline.

These findings are concordant with those of the PROSPECT trial, which demonstrated that preoperative MRI identified occult disease in approximately 11% of patients Stage 1, non-triple-negative breast cancer and was associated with very low early LRR (1%), even among patients in whom RT was omitted [35]. While longer follow-up from PROSPECT is awaited, the results support the clinical relevance of identifying and treating occult disease at diagnosis, consistent with the associations observed in the present study.

This study has several limitations. Its retrospective design introduces potential selection bias and confounding by indication, as CBI use at diagnosis was not randomized and increased over time alongside other improvements in care. Although models were adjusted for key covariates including age, calendar period, stage and MD, residual confounding cannot be excluded. While endocrine therapy recommendation was recorded, data on treatment adherence and duration were not available. Event numbers were relatively small, particularly within subgroup analyses, limiting statistical power and precision of estimates. Follow-up was limited to a median of 4.5 years, and longer-term differences in locoregional events remain unknown; the magnitude and persistence of observed associations may therefore change with extended follow-up, although early differences in locoregional event rates tend to be maintained with more mature follow-up [19, 20]. MD was assessed at the time of first surveillance CEM for all patients to ensure standardised measurement across the cohort. As a result, MD may not fully reflect density at diagnosis, particularly as systemic therapies, may influence mammographic density over time [36]. However, the absence of an observed benefit of CBI in the high MD subgroup suggests that misclassification alone is unlikely to explain the observed association among patients classified as having low MD. Similarly, background parenchymal enhancement (BPE) was only available at the time of surveillance imaging and not at diagnosis for most patients, it was not included in the current analysis but represents a potential area for future investigation. Finally, this was a single-service study with established expertise in both CEM and MRI, which may limit generalisability to other practice settings.

Despite these limitations, this study has several important strengths. First, it represents a large contemporary cohort with over 1,100 women and more than 5,000 person-years of follow-up, providing sufficient power to examine clinically meaningful locoregional outcomes across multiple biologically relevant subgroups. Second, to our knowledge, this is among the first studies to evaluate the association between CBI at diagnosis (including both CEM and MRI) and subsequent locoregional oncological outcomes. Finally, the uniform use of CEM surveillance across the cohort allowed for robust and consistent ascertainment of locoregional events and reduced detection bias related to differential follow-up imaging.

## Conclusion

In this large contemporary cohort, use of CBI for local staging at diagnosis was associated with lower early locoregional event rates among women with invasive breast cancer, particularly those with earlier stage and luminal disease, and with fewer contralateral events. These findings support the concept that more sensitive baseline staging may identify clinically relevant occult disease and reduce early locoregional recurrence. Further prospective studies and longer-term follow-up are needed to better define which patient subgroups derive the greatest benefit from contrast-based staging at diagnosis.

## Supplementary Information

Below is the link to the electronic supplementary material.

Supplementary Material 1

Supplementary Material 2

## Data Availability

The datasets generated during and/or analysed during the current study are not publicly available due to the inclusion of patient-level data containing potentially identifiable information, but are available from the corresponding author on reasonable request, subject to institutional and ethics approval.
