# Supplementary material for: Fewer breast cancers during post-treatment surveillance among patients initially staged using contrast-based imaging
Source: Breast Cancer Res Treat. 2026 Aug 13;218(3):45. doi: 10.1007/s10549-026-08049-6 (PMC13473290; doi:10.1007/s10549-026-08049-6)
Supplement: Supplementary file 2 [file 10549_2026_8049_MOESM2_ESM.docx]

# Supplementary figures and tables

[Supplementary Table 1. Univariate hazard ratios. MD = mammographic density]

|  |  | HR |
| --- | --- | --- |
| CBI | Yes | 0.61 (0.36 – 1.02), p=0.061 |
|  | No | REF |
| Age group | <50 | 1.17 (0.46 – 3.00), p=0.743 |
|  | 50-69 | REF |
|  | 70+ | 0.99 (0.58 – 1.71), p=0.981 |
| period | 2014-2018 | REF |
|  | 2019-2022 | 0.91 (0.54 – 1.51), p=0.704 |
| MD_gp | A/B | REF |
|  | C/D | 1.67 (0.99 – 2.81), p=0.053 |
| Stage | 0 | REF |
|  | 1/2a | 0.43 (0.25 – 0.76), p=0.003 |
|  | 2b/3 | 0.38 (0.15 – 0.96), p=0.039 |
|  |  |  |
| Phenotype group | ER/PR+ HER2+/- | REF |
|  | ER/PR-HER2+/- | 2.11 (1.05 – 4.21), p=0.035 |

[Supplementary Table 2. Time from index diagnosis and detection characteristics of locoregional events]

|  | CBI group (n = 24) | Non-CBI group (n = 36) |
| --- | --- | --- |
| Median time from index diagnosis (months) | 33 | 24.5 |
| Contrast-directed | 13 | 11 |

[Supplementary Table 3. Radiotherapy use by index surgery type and morphology. BCS = breast conserving surgery]

| Group | BCS (N = 903) | Mastectomy (N = 201) |
| --- | --- | --- |
| Invasive | 590/746 | 63/171 |
| DCIS | 78/157 | 0/30 |

[Supplementary Figure 1. Observed locoregional event-free survival for each factor of interest in the adjusted hazards models. MD = mammographic density]

|  |  |
| --- | --- |
|  |  |
|  |  |
